# Diploid wax apple (*Syzygium samarangense*) genome identified NAC genes regulating fruit development

**DOI:** 10.1093/hr/uhae025

**Published:** 2024-01-17

**Authors:** Junyu Zhang, Zhidong Li, Yufan Liang, Shuang He, Guilian Guo, Shuting Yang, Fei Chen, Yinhua Chen, Wenquan Wang

**Affiliations:** College of Breeding and Multiplication (Sanya Institute of Breeding and Multiplication), National Key Laboratory for Tropical Crop Breeding, Hainan University, Sanya 572025, China; College of Tropical Agriculture and Forestry, Hainan University, Danzhou 571737, China; College of Breeding and Multiplication (Sanya Institute of Breeding and Multiplication), National Key Laboratory for Tropical Crop Breeding, Hainan University, Sanya 572025, China; College of Tropical Agriculture and Forestry, Hainan University, Danzhou 571737, China; College of Breeding and Multiplication (Sanya Institute of Breeding and Multiplication), National Key Laboratory for Tropical Crop Breeding, Hainan University, Sanya 572025, China; College of Tropical Agriculture and Forestry, Hainan University, Danzhou 571737, China; College of Breeding and Multiplication (Sanya Institute of Breeding and Multiplication), National Key Laboratory for Tropical Crop Breeding, Hainan University, Sanya 572025, China; College of Tropical Agriculture and Forestry, Hainan University, Danzhou 571737, China; College of Breeding and Multiplication (Sanya Institute of Breeding and Multiplication), National Key Laboratory for Tropical Crop Breeding, Hainan University, Sanya 572025, China; College of Tropical Agriculture and Forestry, Hainan University, Danzhou 571737, China; College of Breeding and Multiplication (Sanya Institute of Breeding and Multiplication), National Key Laboratory for Tropical Crop Breeding, Hainan University, Sanya 572025, China; College of Tropical Agriculture and Forestry, Hainan University, Danzhou 571737, China; College of Breeding and Multiplication (Sanya Institute of Breeding and Multiplication), National Key Laboratory for Tropical Crop Breeding, Hainan University, Sanya 572025, China; College of Tropical Agriculture and Forestry, Hainan University, Danzhou 571737, China; College of Breeding and Multiplication (Sanya Institute of Breeding and Multiplication), National Key Laboratory for Tropical Crop Breeding, Hainan University, Sanya 572025, China; College of Tropical Agriculture and Forestry, Hainan University, Danzhou 571737, China; College of Breeding and Multiplication (Sanya Institute of Breeding and Multiplication), National Key Laboratory for Tropical Crop Breeding, Hainan University, Sanya 572025, China; College of Tropical Agriculture and Forestry, Hainan University, Danzhou 571737, China

Dear Editor,

*Syzygium samarangense*, the wax apple, is a tropical plant species belonging to the eudicot Myrtaceae family. Common names include the wax apple, Java apple, rose apple, and wax jambu. Its native habitat is south Asia and Southeast Asia, and it was introduced to Chinese Taiwan in the 17th century and is now widely cultivated in the tropics. Nowadays it is cultivated in various regions of China [1], including provinces Guangdong, Hainan, and Guangxi. Wax apple is a typical tropical fruit that thrives in warm, humid climates and has a particular fondness for moist and fertile soil. Wax apples, with their high water content and richness in vitamin C, provide excellent hydration benefits for the skin when consumed. Additionally, it contains anthocyanins, which can neutralize harmful free radicals in the body. Wax apples are known for their distinctive aroma and serve as a natural febrifuge. Genome sequences serve as the cornerstone for genetic research and molecular breeding [2]. As of now, there has been no available genome sequence for wax apples.

To address this gap, we chose to investigate the genome of the *S*. *samarangense* cv. ‘Black Sugar Barbilian’, owing to its outstanding performance in the market and its relatively small diploid genome (https://cvalues.science.kew.org). Our goal was to contribute essential genome sequence data and features to foundational research on wax apple because genome sequences are the foundation of genetics and breeding [3–5].

The predicted genome size by flow cytometry is 743 Mb, whereas the estimated genome size by *k*-mer inference is 693.6 Mb (Table 1). Relying on the 115.1-Gb ultra-long reads by Oxford Nanopore Technology, 161.8-Gb DNBSEQ short reads, and 89.8-Gb Hi-C reads, eventually the 676.1-Mb genome spanning 11 chromosomes was assembled for this diploid wax apple. Most of the chromosomes present centromere regions with a high density of repeats and low density of genes (Fig. 1A). The genome size of this species is nearly double that of its close relative, the diploid *S. aromaticum*. This difference can be attributed to the total repeat sequences, which occupy 355 Mb and account for 51.27% of the genome—much larger than the corresponding figures in the clove fruit *S. aromaticum*, where the total repeat sequences amount to 161 Mb, constituting 43.4% of its genome [6]. In *S*. *samarangense*, the total genomic GC content reached 40.87. The genomic Benchmarking Universal Single-Copy Orthologs (BUSCO) value was 98.6% [C: 98.6% (S: 42.1%, D: 56.5%), F: 0.5%, M: 0.9%, n: 2326], suggesting a reference-scale genome for comparative analyses and gene mining. Relying on the 17.2-Gb full-length transcriptome data, we predicted a total of 45 914 protein-coding genes in wax apple (Table 1). We constructed a species tree to show the evolution of wax apple and related species. The wax apple diverged from its relative *S*. *aromaticum* ~15 million years ago (Fig. 1B). These two species shared 15, 433 orthogroups, suggesting they are relatively distinct relatives (Fig. 1C).

**Table 1:** Genomic statistics of wax apple.

**Genome**	***Syzygium samarangense***
Ploidy	2n = 22
Estimated genome size (Mb) *k*-mer	693.6
Assembled genome size (Mb)	676.1
Heterozygosity (%)	1.7
Contig N50 (Mb)	4.62
Number of contigs	293
Number of pseudochromosomes	11
Repeat sequence content (%)	51.27
GC content (%)	40.87
Number of gene models	45,914
Genome BUSCOs (eudicot) (%)	98.6

**Figure 1:**
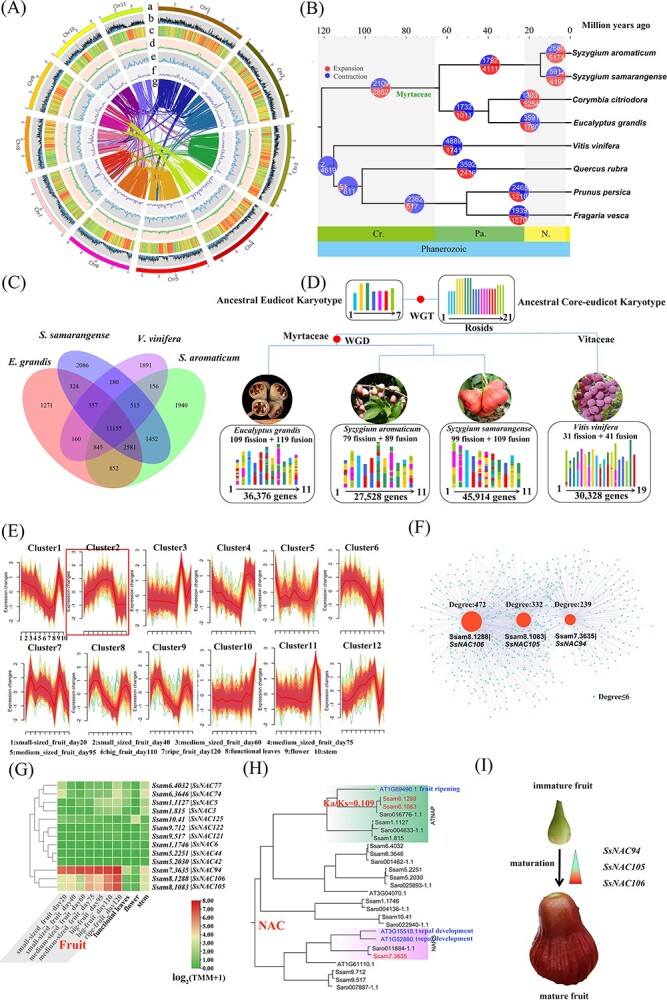
Genome composition and evolution of wax apple fruit. **A** Circos plot showing the genome details. Labels a–g indicate (a) 11 chromosomes of *Syzygium samarangense*; (b) gene density; (c) repeat sequence content; (d) GC content density; (e) density of Copia LTR–RTs; (f) density of Gypsy LTR–RTs; and (g) syntenic blocks (all window sizes = 50 kb). **B** Expansion and loss of gene orthogroups leading to the current wax apple genome. **C** Venn diagram showing orthogroups shared by wax apple and related genomes. **D** Evolution of the karyotypes of wax apple and related genomes. WGD indicates whole-genome duplication; WGT indicates a whole-genome triplication. **E** Wax apple transcriptome analysis unveiled a trend plot illustrating the expression patterns of differentially expressed genes, revealing the presence of 12 clusters. **F** Visualization of the MEblue module revealed three NAC genes with high weighted degrees. **G** Heat map showing the three highly expressed NAC genes. **H** The two close groups of NAC genes are potentially involved in fruit development. **I** We discovered three NAC genes involved in the ripening of wax apple fruits.

We surveyed the genomic duplication of wax apple. We found that the wax apple genome experienced γ whole-genome triplication (WGT) (which is shared by all core eudicots), as well as one recent whole-genome duplication (WGD) shared by the three Myrtaceae family species *S*. *aromaticum*, *S*. *samarangense*, and *Eucalyptus grandis* (Fig. 1D). The γ WGT produced 27 chromosomes for all core eudicots, then 99 fission events and 109 fusion events occurred, leading to the current karyotype composed of 2n = 22 chromosomes.

Wax apple fruit is its most significant economic attribute. In comparison with closely related species within the *Syzygium* genus, such as the clove fruit (*S. aromaticum*), wax apples are known for their enlarged and nutritionally rich berries. Surprisingly, there has been no prior genetic research into this aspect of wax apples. In an effort to unravel essential fruit development genes within the wax apple genome, we conducted transcriptome sequencing of fruit at different developmental stages, as well as control organs, including leaves, flowers, and stems. In this study we explored the core genes associated with fruit maturation. We examined the transcriptomes of wax apple fruits at different developmental stages and in various organs, including small-sized fruit at day 20, small-sized fruit at day 40, medium-sized fruit at day 60, medium-sized fruit at day 75, big fruit at day 95, big fruit at day 110, ripe fruit at day 120, functional leaves, flowers, and stems. We investigated the expression patterns of all genes in these organs (Fig. 1E). Our analysis revealed that all genes in cluster 2 exhibited a significant increase in expression levels during fruit development. Further exploration of cluster 2 identified only five NAC transcription factors: Ssam10.41 (*SsNAC125*), Ssam7.3635 (*SsNAC94*), Ssam8.1288 (*SsNAC106*), Ssam8.1083 (*SsNAC105*), and Ssam11.1329 (*SsNAC149*). To further filter genes related to fruit development, we explored the co-expression network with the R package WGCNA. Genes with consistently low expression across all samples or those with minimal variation (expression value equal to 0) were excluded to construct a weighted gene co-expression network, and we obtained 49 co-expression modules of distinct colors (https://figshare.com/articles/thesis/_/24797238). We conducted a correlation analysis between module features and characteristic traits of fruit development at different stages. Notably, we observed a high correlation between genes in the MEblue module and the developmental stages of wax apple fruits (https://figshare.com/articles/thesis/_/24797238). Subsequently, we visualized the genes in the MEblue module (Fig. 1F), revealing that the top three weighted transcription factor genes were Ssam8.1288 (*SsNAC106*), Ssam8.1083 (*SsNAC105*), and Ssam7.3635 (*SsNAC94*). We conducted an expression analysis of 164 NAC genes in wax apple during fruit development, generating a heat map (https://figshare.com/articles/thesis/_/24797238). Interestingly, we identified a cluster of three NAC genes: Ssam8.1288 (*SsNAC106*), Ssam8.1083 (*SsNAC105*) (Fig. 1G), and Ssam7.3635 (*SsNAC94*), and their expression profiles supported a continuous increase in expression levels throughout fruit development. Phylogenetic analysis also revealed that the three identified wax apple NAC genes share similar functions with homologous genes in *Arabidopsis thaliana*, specifically (Fig. 1H) their involvement in the development of both fruits and sepals. Notably, in wax apple, the fruit primarily consists of modified sepals. This evidence strongly supports the notion that Ssam8.1288 (*SsNAC106*), Ssam8.1083 (*SsNAC105*), and Ssam7.3635 (*SsNAC94*) are indeed key transcription factors associated with fruit ripening development. Their homologous genes in *Arabidopsis* are known to play pivotal roles in the development and maturation of fruit [7]. Hence, it is evident that these three wax apple NAC transcription factors are crucial contributors to the process of fruit maturation (Fig. 1I). Its regulation including the target genes would be an interesting topic in the near future [8].

Overall, our work provides the first diploid genome for the tropical fruit crop wax apple, which could be a reference for genetic studies and lay the foundation for molecular breeding. It will also facilitate comparative genomic studies and help understanding of the evolution of tropical plants and fruit development.

## Acknowledgements

This work was supported by the National Natural Science Foundation of China (32172614), the Hainan Province Science and Technology Special Fund (ZDYF2023XDNY050), and supported by the Project of National Key Laboratory for Tropical Crop Breeding (NO. NKLTCB202337).

## Author contributions

W.W. and F.C. designed this research. J.Z., S.H., S.Y., Y.L., G.G., and F.C. collected the plant samples in spring, summer, and autumn of 2022. J.Z. and Z.L. performed the experiments and analyses. F.C., Y.C., and J.Z. wrote the manuscript.

## Data availability

Raw data Oxford Nanopore Technology sequencing, Illumina, Hi-C data, as well as genome, coding sequences, proteins, and gff files are available online at the National Genomics Data Center (https://ngdc.cncb.ac.cn/). The project ID is PRJCA020470.

## Conflict of interest statement

The authors declare no conflict of interest.
